# Safety by design: Biosafety and biosecurity in the age of synthetic genomics

**DOI:** 10.1016/j.isci.2023.106165

**Published:** 2023-02-10

**Authors:** Stefan A. Hoffmann, James Diggans, Douglas Densmore, Junbiao Dai, Tom Knight, Emily Leproust, Jef D. Boeke, Nicole Wheeler, Yizhi Cai

**Affiliations:** 1 Manchester Institute of Biotechnology, University of Manchester, 131 Princess Street, Manchester M1 7DN, UK; 2 Twist Bioscience, 681 Gateway Boulevard, South San Francisco, CA 9408, USA; 3 Department of Electrical and Computer Engineering, Boston University, 610 Commonwealth Avenue, Boston, MA 02215, USA; 4 CAS Key Laboratory of Quantitative Engineering Biology, Guangdong Provincial Key Laboratory of Synthetic Genomics and Shenzhen Key Laboratory of Synthetic Genomics, Shenzhen Institute of Synthetic Biology, Shenzhen Institute of Advanced Technology, Chinese Academy of Sciences, Shenzhen, China; 5 Ginkgo Bioworks, 27 Drydock Avenue, Boston, MA 02210, USA; 6 Institute for Systems Genetics, and Department of Biochemistry & Molecular Pharmacology, NYU Langone Health, 435 East 30th Street, New York, NY 10016, USA; 7 Department of Biomedical Engineering, NYU Tandon School of Engineering, Brooklyn, NY 11201, USA; 8 Institute of Microbiology and Infection, University of Birmingham, Edgbaston, Birmingham B15 2TT, UK

**Keywords:** Biological sciences, Systems biology, Genomics

## Abstract

Technologies to profoundly engineer biology are becoming increasingly affordable, powerful, and accessible to a widening group of actors. While offering tremendous potential to fuel biological research and the bioeconomy, this development also increases the risk of inadvertent or deliberate creation and dissemination of pathogens. Effective regulatory and technological frameworks need to be developed and deployed to manage these emerging biosafety and biosecurity risks. Here, we review digital and biological approaches of a range of technology readiness levels suited to address these challenges. Digital sequence screening technologies already are used to control access to synthetic DNA of concern. We examine the current state of the art of sequence screening, challenges and future directions, and environmental surveillance for the presence of engineered organisms. As biosafety layer on the organism level, we discuss genetic biocontainment systems that can be used to created host organisms with an intrinsic barrier against unchecked environmental proliferation.

## Graphical abstract

**Figure:**
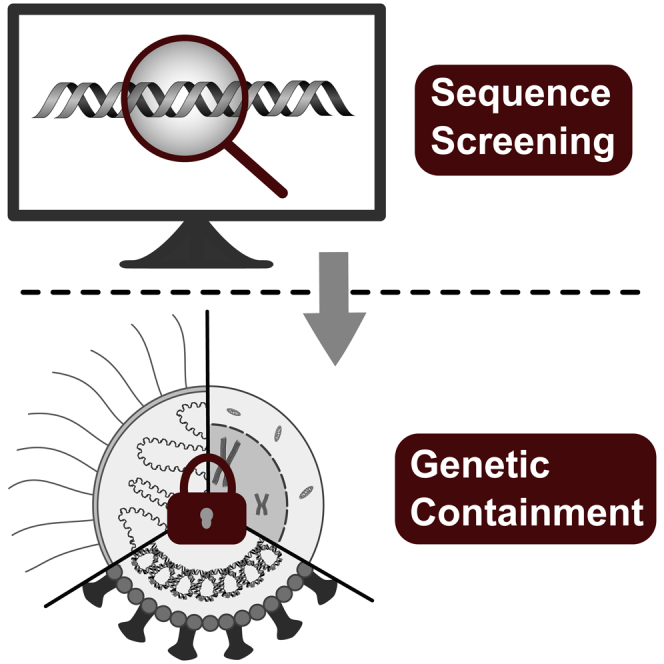

## Introduction

The past decades have seen a tremendous decrease in the cost of DNA synthesis whereas methods for nucleic acid manipulation, assembly and delivery have become more sophisticated and versatile. This has enabled the emergence of the field of synthetic genomics, achieving the total synthesis of viral^1^^,^^2^^,^^3^ and bacterial genomes.^4^^,^^5^ Soon, with the Synthetic Yeast Sc2.0 project^6^ nearing completion, the first synthetic eukaryotic genome will be available. The possibility to create designer genomes provides fundamentally new opportunities to probe and alter biological functions. This will have tremendous economic and societal impacts in biomedicine, biomanufacturing, bioenergy, agriculture, bioconservation and other areas. Around the globe, biofoundries have been established,^7^ enabling unprecedented throughput of assembly and testing of genetic constructs. At the same time DNA ‘printers’, desktop devices producing DNA by enzymatic instead of chemical synthesis, are coming to the market, decentralizing access to synthetic DNA. These devices currently focus on synthesis of shorter sequences (30–80 nucleotides) but the technology has the potential to synthesize sequences of more than 300 nucleotides. Furthermore, ‘do-it-yourself biology’ (DIY BIO) communities have enjoyed increasing popularity, democratizing genetic engineering technology and infrastructure, with limited institutional oversight.

Although these technologies and developments offer vast potential to accelerate biological research and to improve human life through a variety of applications, they also come with inherent risks of accidental or intentional creation and dissemination of potentially harmful biological entities.^8^ Biosafety risks may be posed by unintentional spread of biological agents with the potential to negatively affect human-, agricultural- and ecological health. Special consideration must be given to use cases of engineered organisms in non-contained environments, for example, in medical, agricultural or bioremediation applications. In these cases, it is necessary to ensure that genetically modified microorganisms or engineered genes do not proliferate unchecked. In addition to biosafety concerns, synthetic genomics technologies can pose biosecurity risks because of their potential for dual use. For instance, it is possible to obtain virions solely from sequence information, without requiring actual viral isolates, as demonstrated early on during the ongoing pandemic for SARS-CoV-2,^9^ an RNA virus with a relatively large viral genome. Clearly, this capability can be used to accelerate biological research and to quickly combat global health challenges posed by novel pathogens. However, the same technologies and their relative ease of access can enable malicious state or non-state actors to recreate and engineer dangerous pathogens, with potentially catastrophic consequences. Thus, it is imperative and strategic that safeguarding frameworks and technologies are developed and implemented to safely and securely deliver the revolutionary societal and economic benefits promised by synthetic genomics.

In this article, we review measures and technologies to comprehensively address biosafety and biosecurity risks arising from synthetic genomics capabilities, with a focus on genetically engineered microorganisms (GEMs). We discuss risk mitigation technologies on the digital and the biological layer, DNA sequence screening and genetic biocontainment. Sequence screening aims to control access to genetic material of concern and is already routinely performed by many DNA synthesis providers for orders of gene-length sequences. Genetic biocontainment systems are intended to curb proliferation of GEMs or their DNA outside of specified conditions.

## DNA sequence screening

A clear and immediately feasible intervention point for controlling the emergence of harmful synthetic genes or organisms is the production of DNA used in engineering biology. The 2007 report “Synthetic Genomics: Options for Governance^10^” identified a suite of possible actions by companies, customers, and regulators for preventing biosecurity incidents and concluded that gene and oligonucleotide synthesis companies screening DNA orders was the most effective and tractable action for reducing this risk.

Today, a small number of DNA synthesis firms specialize in synthesizing gene- and pathway-length pieces of double-stranded DNA, which are sometimes incorporated into living cells before shipment to a customer. This industry has been supplying gene-length synthetic DNA to customers for more than 15 years^11^ The need to screen orders placed with these companies was recognized early in the genesis of synthetic biology,^11^ sparking the formation of the International Gene Synthesis Consortium (IGSC), a consortium of gene synthesis companies who have committed to screening DNA synthesis orders and sharing best practices.

### Regulation of access to synthetic DNA

**Figure 1:**
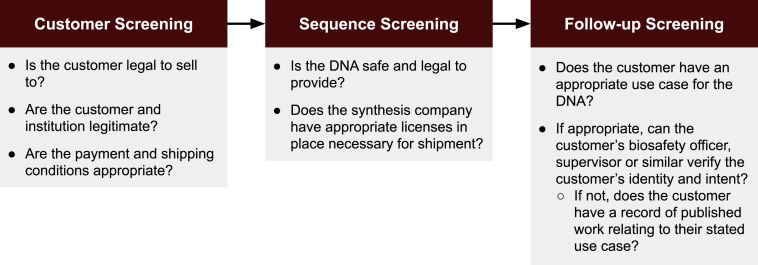
Overview of DNA synthesis screening components

No governments currently require screening or mandate how it is performed, so any companies performing screening of DNA synthesis orders today do so voluntarily. In 2010, the US Department of Health and Human Services (HHS) issued the “Screening Framework Guidance for Providers of Synthetic Double-stranded DNA (https://www.phe.gov/Preparedness/legal/guidance/syndna/Documents/syndna-guidance.pdf)” to provide a high-level framework to DNA providers on how to screen orders. The Guidance states that dsDNA providers should implement customer and sequence screening and perform follow-up screening if the customer or sequence screening raises a concern (Figure 1). In issuing its guidance, HHS defined several key characteristics of each step but allowed flexibility in how organizations implemented this screening. In 2022, HHS released a proposed revision to the guidance (https://www.federalregister.gov/d/2022-09210) that expands the reach of the 2010 guidance to oligonucleotides and a wider range of potentially hazardous sequences. The International Gene Synthesis Consortium (IGSC), a group of gene synthesis providers, published the IGSC “Harmonized Screening Protocol (International Gene Synthesis Consortium. Harmonized Screening Protocol v2.0. 2017. Available: https://genesynthesisconsortium.org/wp-content/uploads/IGSCHarmonizedProtocol11-21-17.pdf)” in 2010. This protocol aligned with the 2010 guidance but is more detailed.

Other countries do not currently have their own guidelines on how to conduct DNA screening, with international companies often referring to the IGSC guidelines to inform screening practices. However, a number of countries are reviewing their biosecurity strategies in the light of the COVID pandemic, for example the United Kingdom, (https://www.gov.uk/government/consultations/biological-security-strategy-call-for-evidence/public-feedback/biological-security-strategy-summary-of-public-response) which has named democratized access to DNA synthesis as key risk area for consideration identified through public consultation. The World Health Organization’s 2022 Global guidance framework for the responsible use of the life sciences (https://www.who.int/publications/i/item/9789240056107) also highlights DNA synthesis as one of its seven illustrative scenarios to illustrate challenges and gaps in the governance of biorisks. The guidance framework recommends DNA synthesis companies screening according to the IGSC’s protocol as a risk mitigation measure to prevent the misuse of synthetic DNA.

**Figure 2:**
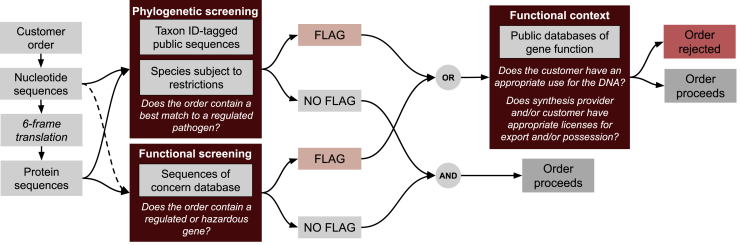
Summary of technical approaches to DNA screening Orders undergo 6-frame translation to retrieve protein sequences. Protein and nucleotide sequences are compared to publicly available protein and nucleotide sequences labeled with the taxonomic ID the sequence originates from, and best matches within the order are identified. Any best matches that correspond to regulated pathogens are flagged if they meet a minimum length requirement for screening (200 bases under 2010 US Government guidance but this threshold can differ from company to company). Sequences are also screened against a database of individual sequences of concern (e.g., controlled toxins). Hits to these sequences are also flagged. Flagged sequences undergo additional follow-up screening, where information available on the flagged sequence’s function is compared with the customer’s stated use case, publication history and institutional affiliation.

An overview of a typical screening workflow is given in Figure 2. The details of screening implementations differ by provider and customer location given the lack of specifics in terms of regulatory requirements. Any orders that are flagged during sequence screening must undergo additional screening to assess the customer’s intended use, the ability to perform the described work and the biological function of the ordered DNA. This follow-up investigation is the most time-consuming step in DNA synthesis screening. It typically requires a staff member with a PhD in bioinformatics or similar training to perform the work. Follow-up screening may also involve contacting the relevant regulatory authorities, such as the Bureau of Industry and Security in the US Commerce Department, to confirm whether a specific sequence is subject to an export control license requirement or not. As the cost of DNA synthesis drops and demand increases, this manual evaluation of orders is becoming less practical, creating a need for automatable approaches.

### Technical approaches to screening

IGSC members screen orders against a Regulated Pathogen Database, assembled and curated by the IGSC to include data from all organisms on the US Select Agent and Toxin list (https://www.selectagents.gov/sat/list.htm), the Australia Group Common Control List (https://www.dfat.gov.au/publications/minisite/theaustraliagroupnet/site/en/controllists.html), and other national lists of regulated pathogens and toxins. These lists cover internationally recognized threat organisms and viruses (such as *Variola major*, the virus that causes smallpox, or *Bacillus anthracis*, the causative agent of anthrax) and toxins like tetrodotoxin. Members screen a six-frame translation of each order to evaluate encoded biological functions rather than just DNA sequence identity.

Although the core goal of sequence screening is to determine whether an ordered sequence could be used to do harm,^12^ current regulatory controls focus on orders that contain sequence that is unique to regulated viruses or toxins or to genes that ‘endow or enhance’ pathogenicity from regulated bacteria, fungi and parasites. These restrictions often include exemptions for specified vaccine strains or inactivated/inviable forms of these pathogens (https://www.ecfr.gov/current/title-42/chapter-I/subchapter-F/part-73).

A major challenge posed by current screening tools is a high false-positive rate - benign sequences are flagged for manual review too frequently.^12^ A common source of false positives are so-called ‘housekeeping genes’ from regulated pathogens (genes not directly involved in pathogenesis, although some believe these should still be considered sequences of concern^13^). In addition to clear false positives, there are also ambiguities about which functions should be viewed as hazardous and thus have their access restricted. The genomic era has revealed that many virulence factors can be found in non-pathogenic bacteria, particularly those involved in interaction with a host.^14^ For example, in *Escherichia coli*, pathogenicity islands coding for fimbrial adhesins can be detected in commensal strains of the normal intestinal microflora. The “Best Match” criteria recommended in the 2010 HHS Guidance is intended to address this problem at least partially by ensuring that only sequences unique to regulated pathogens fall under regulatory controls.

In addition to those sequences subject to formal regulatory control, there is a broader set of sequences often referred to as ‘sequences of concern’ – sequences that might be subject to some form of misuse but that do not pose sufficient danger to merit regulatory control. Such sequences can sometimes cause similar although less-severe illnesses.^15^ DNA synthesis companies screen to ensure customers will use DNA products responsibly and detecting such sequences of concern, while not legally mandated by any government, falls squarely within this goal. There are also limitations to these systems – DNA synthesis companies do not generally have access to the broader context for use of a sequence or how multiple sequences may act in concert in a specific chassis organism.^16^

Current HHS guidelines specify that regions of 200 bases or more that are a best match to a regulated gene or pathogen should be flagged for further attention, so many methods restrict screening and analysis of hits to regions of this length or longer. Local sequence alignment algorithms (e.g., BLAST) are still the most popular for screening orders, partly because flagged hits are easily interpretable. However, these approaches are slower than newer, k-*mer*-based methods that count exact matches between short fragments of defined length *k* (called k-mers) without performing an alignment step, to gain an approximation of sequence identity within a much shorter time frame. Matches between all k-mers can be calculated, or a ‘sketch’ can be computed for each sequence, which retains a defined number of the lexicographically smallest kmers from the full set of observed k-mers in a sequence (https://genomebiology.biomedcentral.com/articles/10.1186/s13059-016-0997-x). K-mer similarities between sequences, however, degrade with increasing sequence divergence much faster than alignment-based sequence similarity (e.g., for k = 31, a popular choice, sequences below 80% nucleotide identity cannot be detected),^17^ creating potential sensitivity problems.

**Existing screening solutions**

The Black List Sequence Screening Pipeline (**BLiSS**) (https://www.aiche.org/sbe/conferences/synthetic-biology-engineering-evolution-design-seed/2016/proceeding/paper/bliss-black-list-sequence-screening-pipeline) was developed by the U.S. Department of Energy Joint Genome Institute with guidance from the US Department of Health and Human Services. BLiSS detects “sequences of concern” of at least 200 nucleotides in length. A “Best Match” approach is used to determine whether any of the sequences of concern are unique to Select Agents or Toxins, or CCL-listed agents, toxins or genetic elements. All hits are broken up using a 200 bp sliding window to determine the Best Match for each fragment. The pipeline was adapted using over 10,000 sequences submitted to the JGI for synthesis to reduce false positives.

**SeqScreen**^18^ was designed to sensitively assign taxonomic classifications, functional annotations, and biological processes of interest to short nucleotide sequences of unknown origin (40 bp-1,000 bp). An open-source solution, it is not explicitly designed for identifying biosecurity threats, but can be used for this purpose. Highly trained individuals read a vast amount of literature to provide accurate annotations for each sequence in their database of concern. The teams are exploring machine learning approaches as a way of automating a portion of this work by predicting a suite of biological processes of interest on large protein databases before incorporation into the SeqScreen framework.

**ThreatSeq** (https://www.battelle.org/markets/health/chemical-and-biological-threats/biosecurity-pandemic-preparedness/threatseq) is a commercial solution from Batelle for screening synthetic DNA orders that identifies similarity to specific sequences of concern and Select Agent genomes. They use a tiered reporting style to reflect different levels of risk posed by different orders and the scientific uncertainty around the relevance of different sequences to pathogenicity. ThreatSeq’s database currently contains more than 10,000 Sequences of Concern, which are constantly under review and encompass all regulated organisms, viruses and toxins. The ThreatSeq metadata is the product of several years of government-funded curation efforts, an indicator of the high cost required to produce a set of annotated sequence records.

**SecureDNA** (https://www.securedna.org/) differs from previous approaches by using a one-way hash algorithm to create digests of sequence regions to obscure customer orders from the screening provider and Sequences of Concern from both customers and DNA providers. The database contains short strings of numbers and letters (a digest) produced by a hash function from k-mers of both protein and nucleotide sequences. These k-mers are drawn from conserved regions of Sequences of Concern and mutated variants predicted to preserve the biological function of the original sequence. This digest-based approach allows the inclusion of sequences of concern without revealing their identity.

The Nuclear Threat Initiative is developing an **International Common Mechanism** (https://www.nti.org/analysis/articles/common-mechanism-prevent-illicit-gene-synthesis/) for DNA synthesis screening in consultation with an international technical consortium of industry, policy and academic stakeholders. The Common Mechanism is intended to act as a common standard for screening worldwide and a baseline capability that DNA providers can adopt. This platform identifies genes of concern and best matches to regulated pathogens. It has been designed to accommodate different lists of controlled organisms and provides customizable reporting according to individual regions’ legal requirements.

### The path forward for DNA synthesis screening

As DNA synthesis advances, the cost of producing DNA has gone down (http://www.synthesis.cc/synthesis/category/Carlson+Curves) but the cost of performing screening has not. Without a legal requirement for screening (which no country has yet imposed), firms place themselves at an economic disadvantage if they choose to invest in- and perform routine screening. This disincentive must be addressed if screening is to remain an economically viable practice for competitive businesses.^19^ Options include improving screening to reduce false positives, outsourcing follow-up investigation to specialized vendors, financially compensating companies for screening,^19^ or making screening a legal requirement.^20^ This last option, however, would require broad international adoption to ensure a level economic playing field for the deeply international DNA synthesis market.

In terms of knowing whether DNA synthesis screening is accurately detecting sequences of concern, there are no broadly available standardized performance benchmarks that screening software must meet to demonstrate that the software is performing well. Having regular benchmarks of available tools and well-designed test sets would help evaluate whether new offerings are fit for purpose and to monitor whether existing tools remain robust to growing reference sequence databases and changing regulatory controls.^12^ Test datasets would need to be updated regularly to prevent over-training of screening algorithms on a specific test set.

The bulk (by number of unique ordered sequences) of the synthetic DNA (and RNA) market is for shorter-length oligonucleotide constructs (less than 200 bp in length). Previously, industry-wide screening of oligonucleotide orders was considered intractable because of the far greater volume of companies and orders in this market, the faster turnaround time expected by customers, and the higher false-positive rate of screening shorter sequences.^19^ However, the newly proposed HHS guidance has expanded recommendations for screening to low-volume orders of sequences 50 bases or longer and high-mass orders (>1 mM) of 20 bases or longer. The screening of these shorter sequence lengths poses a new technical challenge, as the probability of spurious similarity between orders and Sequences of Concern increases as sequence length decreases.

In addition to commercial providers of synthesized DNA, some firms sell benchtop DNA synthesizers for individual laboratory use. The development of these technologies is now reaching some crucial milestones, which increases the urgency of universalizing screening. First, the accuracy of DNA synthesis by benchtop devices is anticipated to improve substantially (https://www.nature.com/articles/s41587-020-0695-9); second, companies are now producing DNA of length ∼300 bases.^21^ As a result, there is growing interest in requirements to screen customers buying these devices, screen the sequences produced by these devices for potential hazards, and log the synthesized sequences (https://www.jcvi.org/sites/default/files/assets/projects/dna-synthesis-and-biosecurity/report-complete.pdf).

Current sequence screening is predominantly based on DNA derived from control lists of organisms of concern rather than genes or biological functions of interest/concern.^11^ There is interest in moving away from this agent-based screening toward function-oriented screening for Sequences of Concern that could pose a serious risk if misused.^16^ Virulence factor databases could provide important candidates for Sequences of Concern.^22^ However, the use of these databases has limitations. First, these databases often focus on common human pathogens, which are not the only high-priority pathogens from a biosecurity perspective. Second, most virulence factors can also be found in non-pathogens,^14^ raising uncertainty about whether they should be flagged as hazards and if it is practical to conduct follow-up screening on these orders. Third, many virulence factors presumably remain undiscovered. A recent effort to define Functions of Sequences of Concern (FunSOCs)^23^ may address some of the uncertainty around which virulence factors constitute intrinsic hazards and which do not. However the best way to identify and catalog these is still a matter of debate, as this potentially poses an information hazard.^24^

Another potential future direction of DNA screening is the adoption of screening earlier in the process of ordering DNA, such as in synthetic biology design software, or as part of institutional requisition systems, allowing institutional biosafety officers to submit pre-approval of sequences with DNA synthesis orders to expedite screening. Technical challenges arise in ensuring that screening is used in practice and updated regularly, but implementing screening at these earlier stages could result in a lower burden of follow-up screening for DNA providers and ensure that customers receive their orders without unnecessary delay.

### Environmental surveillance

Sequence screening plays an important role in restricting access to DNA of concern, but it also offers the potential to detect biological threats through metagenomic analysis of environmental samples. Wastewater metagenomics has been successfully employed in the surveillance of a number of infectious diseases and public health threats,^25^ including COVID-19.^26^ Samples are relatively accessible compared to clinical samples, and are culture-independent, meaning pathogens with strict growth requirements (e.g., viruses that only replicate in human cells) can still be detected. Metagenomics can be expensive and technically challenging, but there is an opportunity to leverage this surveillance to serve a host of purposes, including monitoring for the release of engineered organisms.

The depth of sequencing required to correctly distinguish engineered and non-engineered organisms is not currently known, as genetic engineering detection and attribution is typically performed on known sequences or amplified pre-selected markers of genetic engineering. Given sufficient data coverage, sequence data could be fed into algorithms trained to recognize engineered DNA and even to attribute these engineered organisms to a lab of origin.^27^ It is unknown how these methods would perform if a laboratory changed the organism they were working on to engineer a hazard. It is also unknown how these methods would perform on incomplete sequences or even mixed metagenomic samples.

For environmental metagenomics to be most effective, long-term funding and implementation will be needed to establish baseline rates and diversity of genetic markers of engineering in the environment, track trends and identify disruptions in these patterns. This is particularly important for machine learning-based methods that are typically only trained on engineered sequences and do not disclose what they are using to flag engineering. Established surveillance for GEMs in the environment would be able to act as a warning system for potential biosafety and biosecurity threats emanating from non-sanctioned releases of engineered organisms.

## Genetic safeguards

Clearly, it is vital to curb known and unknown risks to public health, agriculture and ecology, arising from the inadvertent or deliberate release of GEMs into natural environments. The measures outlined above aim to control access to DNA sequences of concern and detect engineered organisms in environmental samples. Effective and reliable ways to actually prevent unwanted proliferation of GEMs or their recombinant DNA in natural environments are another important pillar of biosafety. Solving this question is imperative to gain regulatory and public acceptance of open-environment applications of engineered microorganisms, e.g., for bioremediation, agricultural or medical purposes. Although methods of physical containment for such scenarios, for instance through microencapsulation,^28^ have been proposed, we will focus on in-built genetic features that prevent survival of engineered organisms outside of defined environments and/or sexual or horizontal transfer of genetic material to natural organisms.

Genetic safeguard systems acting on the organism level are typically designed to limit survival to permissive environments characterized by the availability or absence of certain compound(s). Under these permissive conditions a wild-type like fitness is highly desirable to not create evolutionary pressure favoring loss of the genetic biocontainment system. Owing to the potential for quick replication of microorganisms, even slight fitness advantages gained by escape mutations will eventually take over in populations of GEMs under permissive conditions, voiding the genetic control system. If the survival conditions are not met, constituting a restrictive environment, the deployed system is supposed to robustly prevent proliferation and optimally induce death of the engineered organisms.

According to the NIH Guidelines for Research Involving Recombinant or Synthetic Nucleic Acid Molecules (https://osp.od.nih.gov/wp-content/uploads/NIH_Guidelines.pdf), survival of the organism or escape of the recombinant DNA should be less than 10^−8^ for contained host-vector systems (EK2). Considering that a single test tube of densely grown *E. coli* easily contains 10^9^ cells, this degree of stringency is insufficient for open-environment uses of biological entities with self-replicative potential. The combination of several orthogonal genetic containment systems should allow containment stringencies several orders of magnitude higher. Also, effective containment is expected to be maintained far longer, as evolutionary enrichment of escape mutations against one of the systems can be prevented through redundancy of containment with additional orthogonal systems. Here, genetic biocontainment systems of a wide range of technology readiness levels are discussed (Table 1).

**Table 1:** Major groups of genetic biocontainment systems

Containment system	Advantages	Disadvantages
Gene knockouts creating auxotrophies	• Easy to implement • Resistant against mutational escape	• Required natural metabolite can be available in natural environments e.g., by cross-feeding
De-repression of suicide genes	• Large available repertoire of suitable effectors • Coupling to environmental sensing possible	• Prone to mutational inactivation of effector gene, exacerbated by incomplete effector repression
Control of essential genes	• Control approaches along the flow of genetic information can be combined • Coupling to environmental sensing possible • Largely resistant against mutational escape	• Typically, small number of essential genes suitable, large-scale screenings necessary for their identification
Orthogonal central dogma	• Both semantic and trophic barrier to natural organisms possible • Highly resistant against mutational escape	• Difficult to develop and implement

### Gene knockouts

The necessity of working with safe organisms was already recognized in the early days of genetic engineering. Just a few years after the first animal gene had been cloned in 1973, the *E. coli* strain χ1776 was introduced.^29^ It was highly sensitive against bile and dependent on availability of thymine/thymidine and diaminopimelic acid, rendering it incapable of colonizing the guts of mammals even in the absence of competing microbes.^30^ Despite even being employed in industrial settings, its use was eventually ceased in favor of strains that were easier to work with.

However, the containment approach of using gene knockouts for reduction of competitive fitness or creation of metabolic deficiencies has been widely used since. Many currently used laboratory strains have such genetic features. For instance, *E. coli* K-12 derived laboratory strains typically feature a *relA* mutation, reducing starved survival,^31^ and often also possess one or multiple auxotrophies, the inability to produce essential metabolites. Likewise, the commonly used *Saccharomyces cerevisiae* lab strains BY4743 and W303 and their haploids feature a number of auxotrophies. These metabolic deficiencies are routinely leveraged in the lab by using complementing genes as selective markers for plasmid maintenance or genomic integration. They also decrease competitive fitness under dynamically changing conditions and nutrient limitations^32^ which are typical for natural environments. Auxotrophic containment has been employed in applied medical research.^33^ *Lactococcus lactis* has been engineered for intestinal delivery of human interleukin 10 to combat inflammatory bowel disease. By replacing its thymidylate synthase gene with the human *IL10* gene, insertion of the transgene rendered it auxotroph for thymidine. Knockout-based auxotrophies are likely the most commonly used genetic biosafety feature currently, albeit not a universally secure one. The nucleic acids, vitamins or amino acids that organisms have been made dependent on may be readily available in some natural environments, e.g., by cross-feeding from other organisms.

A combination of knockouts of endogenous phosphate transporters and introduction of two heterologous genes for transport and metabolization of phosphite has been used to make cyanobacteria dependent on phosphite as a phosphorus source.^34^ This metabolic bypass both restricts the engineered organisms’ survival to controlled environments offering phosphite, and offers a means to preclude contamination with natural, phosphate-dependent organisms if phosphite is the only source of phosphorus.

As was already shown with χ1776 and bile, knockouts can also be used to make microorganisms susceptible to certain environmental conditions instead of creating dependence on exogenous supply of a metabolite. A recent example is the knockout of fluoride exporters in yeast, resulting in sensitivity to fluoride at concentrations acceptable in drinking water.^35^ Because they rely on gene knockouts, these containment strategies are thought to be evolutionarily robust, requiring a mutational gain of function for escape. However, the specific environmental requirements of restrictive conditions mean these strategies cannot be employed as a generic biocontainment method against survival in natural environments.

Clearly, for safe open-environment uses of engineered microorganisms there is a need for specifically designed containment systems to make survival dependent on the availability of a select compound, e.g., one generally not found in natural environments. This need has been recognized and in the past decades notable research into dedicated genetic systems has been conducted. These active containment systems typically confine survival by using lethal factors and/or by regulating the activity of essential genes.

### Suicide genes

The use of lethal genetic factors, or “suicide genes”, as effectors in active biocontainment systems has been a prominent strategy, particularly in prokaryotes. This is largely thanks to the number of discovered prokaryotic toxin-antitoxin systems, giving researchers a repertoire of effective toxic genes for construction of suicide systems in bacteria. In proof-of-concept studies, toxin genes were driven by inducible promoters.^36^^,^^37^ Conditions leading to de-repression of the promoter constitute restrictive environments, causing toxin expression and cell death. Closer to the potential use case of bioremediation, similar suicide systems were extended with a sensing system for a chemical contaminant to be removed, triggered when the chemical is depleted. Notable examples^38^^,^^39^^,^^40^ made use of a promoter inducible by benzoic acid derivatives. Using prokaryotic toxin-antitoxin systems has the advantage that the cognate toxin-antitoxin interaction can be leveraged for toxin control.^41^ This has also been used for a plasmid biocontainment system engineering specific host-plasmid dependence.^42^ A list of toxin-antitoxin systems suitable for biocontainment has been compiled and reviewed elsewhere.^43^

*Cas* proteins have been used as an efficient killing effector in both prokaryotes and eukaryotes.^44^^,^^45^^,^^46^ Used with guide RNAs targeting repetitive regions, a high toxicity results from the numerous induced double-strand breaks. The bipartite nature of the system, requiring both the Cas9 protein and the guide RNA, allows control over expression of both and evidently affords very low basal toxicity.

With suicide-based systems, killing can be impaired by loss of function of the suicide gene or acquired resistance. Basal expression of the suicide gene evolutionarily promotes these escapes. Consequently, stringent control of the suicide gene expression is paramount and, for instance, has been implemented by bifurcated control of suicide gene expression.^40^ However, even sophisticated multi-layered suicide circuits with stringent control of suicide gene expression can suffer from increasing escape frequency by mutational inactivation, as evidenced for the so-called Deadman kill switch within a few days of passaging.^47^

### Control of essential genes

**Figure 3:**
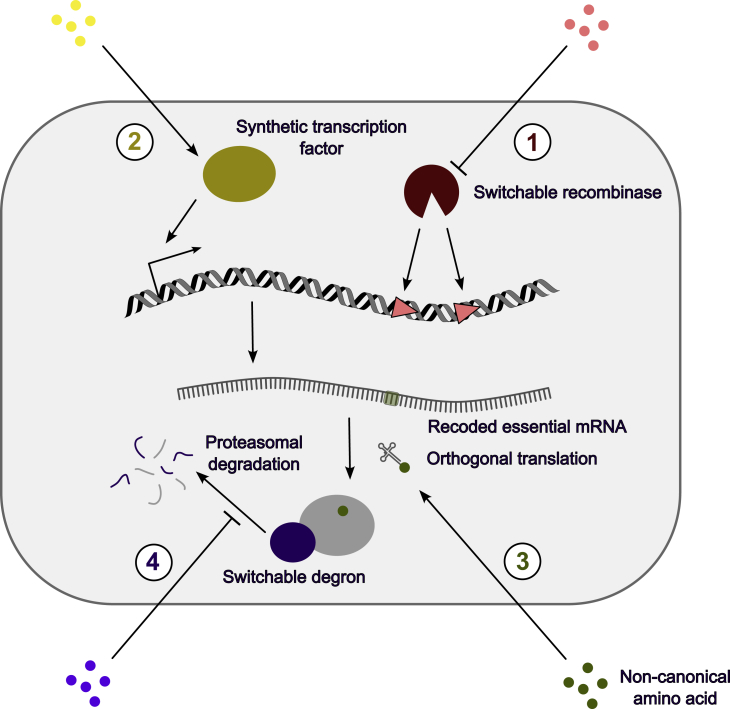
Biocontainment by control over essential genes Control can be attained by independent systems at different points along the flow of genetic information, allowing the free combination of different systems. (1) Recombinational switches lead to the excision of essential genes in restrictive conditions. (2) Transcriptional switches drive expression of essential proteins only if specific chemical inducers are available. (3) Post-translational switches direct essential proteins for degradation, leading to cell death unless a ligand is supplied. (4) Genetic code expansion can be leveraged to make cell survival dependent on the exogenous supply of a non-canonical amino acid.

Controlling essential genes is a potentially more mutation-resistant containment approach; unlike suicide genes, biocontainment escape is usually not possible by easily obtainable loss-of-function mutations. This control can be achieved in a number of ways—notably by making transcription or translation of essential genes, or the stability or function of essential proteins conditional (Figure 3). Another possibility is the conditional removal of essential genes, for instance by an estradiol-activated Cre recombinase fusion protein.^48^ In this approach, the recombinase effectively acts as a ‘suicide gene’.

Conditional transcription of essential genes has been demonstrated as a viable approach for biocontainment in yeast.^48^^,^^49^ In this particular system suitable essential genes are under the control of *GAL* promoters. The chimeric transcriptional activator GEV^50^ drives expression from these promoters only in the presence of estradiol. The cells are thus strictly dependent on the presence of either galactose or estradiol. Controlling the expression of an essential histone gene in this way yielded an escape rate of 10^−6^. Exerting the control on two histone genes reduced this rate to as low as 10^−9^, but resulted in a significant fitness defect under permissive conditions.^48^ A systematic search for more suitable control targets from 250 essential genes yielded three essential genes giving escape rates of less than 10^−7^ individually with minimal changes to the transcriptome.^49^ There is a comparable yeast chimeric transcription factor using zinc finger DNA-binding domains, which activates transcription from a synthetic promoter in the presence of estradiol (ZEV).^51^ In contrast to GEV, ZEV has no apparent off-target effects and therefore was also investigated by Agmon et al. for the three top hits from their screening. Recently, the ZEV system was applied to 1,022 (of ∼1,100) essential yeast genes, and the growth profile in a range of estradiol concentrations was assessed for each.^52^ This screen yielded hundreds of essential genes resulting in estradiol dependent growth under GEV control, providing a resource to identify essential genes suitable for biocontainment leveraging this system. Another attractive feature of this system is that nanomolar concentrations of estradiol required add minimal cost to fermentations, and this low abundance also means they will be difficult to detect by potential bad actors seeking to bypass these containment systems.

Control over translation of essential genes can be obtained in different ways. One developed approach makes use of well-established allosteric transcription factors LacI and TetR for transcriptional control over riboregulators, which in turn affect translation of the target essential gene.^53^ Another method leverages orthogonal translation systems (OTSs) selectively incorporating non-canonical amino acids at repurposed stop codons. An OTS consists of two components, an aminoacyl-tRNA synthetase and a cognate tRNA, which are orthogonal to the endogenous tRNAs and synthetases. The tRNA, loaded with the non-canonical amino acid by the orthogonal aminoacyl-tRNA, has an anticodon recognizing the repurposed stop codon, leading to ribosomal incorporation of the non-canonical amino acid instead of a translational stop. A stop codon reassigned in this way is introduced into an essential gene, such that translation of the functional full-length protein requires presence of the non-canonical amino acid. In the absence of the non-canonical amino acid a truncated, non-functional gene product is being made, resulting in cell death. This approach, creating “synthetic auxotrophy” for the non-canonical amino acid, has been demonstrated in a highly engineered *E. coli* strain devoid of TAG stop codons and release factor 1 (RF1). The introduction of two to three TAG stops at carefully chosen essential gene positions and optimization of the OTS^54^ or the genomic background^55^ resulted in escape frequencies <10^−11^ in non-permissive medium, constituting an extremely tight control of cell survival. The impending completion of the first synthetic eukaryotic genome in the Sc2.0 project will yield an *S. cerevisiae* strain without TAG stop codons. This provides an opportunity to develop highly stringent OTS-based biocontainment in yeast. Furthermore, the same approach can be used to prevent horizontal gene transfer of genes of interest: by introduction of reassigned stop codons into these genes, they will not yield functional gene products when introduced into natural organisms.

Besides controlling transcription and translation of essential genes, the stability of the essential proteins themselves can be made conditional. This can be achieved by fusing them to regulatable degrons. Conditional survival by fusing suitable degrons to essential proteins in yeast has been demonstrated with the auxin inducible degron (AID),^56^^,^^57^ a protein degradation system taken from plants, and with the SMASh tag, a self-cleaving degron whose removal can be inhibited by a small molecule.^58^ In the case of the AID, the tag that is fused to the protein of interest is fairly small, but the system requires heterologous expression of the TIR1 protein, which is not found in non-plant eukaryotic cells. Addition of auxin leads to polyubiquitination of the tag, marking it for proteasomal degradation. With SMASh, the tag consists of the degron itself at the distal end to the fusion, a protease from hepatitis C virus, and a cognate protease cleavage site. Self-cleavage of the tag leads to its removal, leaving behind only part of the cleavage site. Addition of a specific protease inhibitor prevents the self-removal of the tag, leading to protein degradation because of the attached degron. Although these studies show that the survival of microorganisms can be controlled by conditional stability of essential proteins, the operational logic of both systems may not be suitable for typical biocontainment applications with this approach, though: the addition of specific small molecules, rather than their absence, leads to depletion of the tagged essential protein and thus cell death. Another class of switchable degrons called destabilizing domains (DD)^59^ might make it possible to leverage conditional essential protein stability for a practical biocontainment system. These are protein domains engineered to be unstable in the absence of their ligand and thus degraded with a protein they are fused to. Addition of the ligand leads to domain stabilization, preventing protein depletion. With DDs being comparably large tags, effects on protein expression and function are to be expected, likely making it necessary to carefully choose targeted essential genes to get stringent containment without reducing fitness under permissive conditions.

Finally, the function of essential proteins can be a potential target for exerting control over organism survival. A previously described containment system inspired by a protein engineering approach creating *de novo* allostery in enzymes, making enzyme activity dependent on the availability of a ligand.^60^ Using directed evolution, five essential enzymes in *E. coli* have been engineered to require a ligand for their activity.^61^ Combining three engineered, benzothiazole dependent enzymes in a single strain gave an escape frequency of <3∗10^−11^ over two days, clearly making this a promising approach.

### Orthogonal central dogma

Instead of engineering individual safeguarding systems, globally altering the genetic code or the information carrier altogether can create a powerful barrier to genetic interaction with natural organisms. This protection against horizontal gene transfer owing to genetic code incompatibility is referred to as semantic biocontainment. The alteration can also make the organism dependent on synthetic compounds, creating a synthetic auxotrophy, i.e., trophic biocontainment.

Engineering a genetic code that is incompatible to natural organisms is a conceptually attractive way to curb risks arising from genetic exchange of GMOs with the natural environment. Substantially altering the way mRNA messages are decoded into proteins means that any genetic material exchanged between natural and synthetic organisms will not yield functional proteins. A cell-free translation study swapping serine, leucine and alanine codons found functional protein exclusively expressed if the genetic code of mRNA and tRNAs was matched, demonstrating the potential to create a powerful genetic separation to natural organisms.^62^ However, creating an organism with sense codon reassignment is a daunting task considering the vast biochemical, thermodynamic, and semantic complexity and interconnectedness of translation. For instance, the same *in vitro* study reported lowered protein expression from re-coded mRNA by matched translation systems, probably because of altered mRNA folding.^62^ Accordingly, a similarly profound change of an organism’s genetic code would likely require optimization of codon usage in hundreds of essential genes.^63^ Still, there are abundant examples of non-standard genetic codes in nature.^64^ In fact, eukaryotes across a wide range of phylogenetic groups possess mitochondrial genetic codes deviating from the standard code in different ways. The comparably small number of proteins encoded by mitochondrial genomes likely constitutes a much smaller evolutionary barrier to changes of the genetic code. Non-standard nuclear codes do not appear to be as diverse, and most of these aberrant codes have a reassignment of a stop codon to a sense codon. However, some *Candida* yeast species decode a canonical leucine codon as a serine, a change that evolutionarily likely was preceded by code ambiguity.^65^ This mechanism could possibly be used to evolve sense codon reassignments by a stepwise tRNA dosage change from one isoacceptor class to another. Another path to sense codon reassignment is through codon compression followed by codon reassignment. Using a synthetic genomics bottom-up approach, a viable *E. coli* strain free of two serine codons and the amber stop codon, Syn61, has recently been created.^5^ This enabled the deletion of the respective decoding tRNAs and release factor 1, conferring immunity to bacteriophages.^66^ The freed-up codons were subsequently reassigned with orthogonal translation systems to encode non-canonical amino acids. Likewise, reassignment to endogenous amino acids can be used to create two-way barrier to genetic interaction with natural organisms.^67^ However, there are constraints surrounding tRNA crosstalk and the dependence of many aminoacyl-tRNA synthetases on the anticodon for tRNA recognition. Thistype of recoding may find itself in tension with the goals of biosecurity screening of synthetic DNA. Synthetic DNA designed for use in organisms using alternative genetic codes, when translated using standard genetic codes, would not be identifiable as Sequences of Concern even if they encoded such a sequence in the target non-natural genetic code.

To achieve genetic code incompatibility, instead of reassigning existing codons, entirely new codons can be created. This possibility has been demonstrated by systems specifically decoding quadruplet instead of natural triplet codons.^68^^,^^69^ Another avenue for codon creation is the expansion of DNA polymer chemistry using non-natural base pairs. A few groups have managed to create unnatural DNA base pairs in DNA polymers, which are faithfully replicated in PCR reactions and can be transcribed into RNA *in vitro*.^70^^,^^71^ The Romesberg group has succeeded in maintaining an unnatural base pair (dNaM-dTPT3) in *E. coli*^72^ and reported translation of mRNA and tRNA containing unnatural base pairs, enabling ribosomal decoding of codons with unnatural bases.^73^ Still, achieving DNA replication, transcription and translation of unnatural base pairs as efficiently and faithfully as natural base pairs currently appears to be a distant feat. Once achieved, it will open up a powerful way to create semantic biocontainment and synthetic auxotrophy.

An even more ambitious undertaking is altering the central dogma altogether by using an alternative information-carrying polymer. Several non-natural nucleic acid backbone chemistries have been demonstrated to be suited for information storage, and polymerases have been engineered both to specifically synthesize these XNAs (xeno nucleic acids) from DNA templates and to transcribe XNAs back to DNA.^74^ DNA could be produced in *in vivo* by the cellular machinery of *E. coli* from different non-canonical backbones.^75^^,^^76^ Whether XNAs can take on functions of and potentially replace natural nucleic acids in living cells remains yet to be seen. The thought of synthetic organisms with a different central dogma is certainly enticing, not least from a biocontainment point of view.^77^

## Conclusion

With increasing affordability of DNA synthesis and rapid technological advances in synthetic genomics, an ever-growing number of actors are capable of engineering or building genomes. The nascent decentralized capabilities of creating designer genomes challenge existing biosecurity and biosafety frameworks, which need to be adapted and updated to address these challenges.

Because synthetic genomics technologies can remove the need for biological isolates to create certain pathogens, restricting access to synthetic DNA of concern is a major safeguard against its harmful use. Already widely implemented measures of customer and sequence screening discussed above are largely a result of industry self-regulation. Notably, however, sequence screening has an economic cost, e.g., compiling and maintaining an extensive database of sequences of concern and suitable screening algorithms with the computational costs necessary to perform these screens. Furthermore, sensitive detection comes with trade-offs relating to computational expense and false positive hit rate with associated costs of manual follow-up procedures. These costs provide an economic disincentive for synthesis companies to implement rigorous screening procedures, particularly for smaller companies under legislations with little regulation of the synthetic DNA market. The Nuclear Threat Initiative has proposed to develop a common screening mechanism, providing both a comprehensive database and screening algorithms, to partially externalize the costs of sequence screening. Reducing these economic disincentives should be complemented by coordinated regulation of the global synthetic DNA market, perhaps as part of existing multilateral arrangements like the Australia Group. Regulating synthetic DNA, however, comes with challenges not encountered with ‘conventional’ biological agents. Sequence screening algorithms can potentially be gamed and do not generally have sufficient awareness of the broader customer goals across sequence providers and orders (for instance, in the way synthetic DNA is (re-)assembled). The emergence of desktop devices enabling decentralized DNA synthesis stresses the question of whether sequence screening should be done locally or centrally, and how to prevent attempts at circumventing oversight. Clearly, developing scalable solutions that incentivize effective control of synthetic sequences of concern while balancing the needs of the various actors and stakeholders is not trivial.

A related issue is that of ensuring biosafety of physically created (partially) synthetic organisms, particularly organisms that contain sequences of concern or are intended for applications in open environments. For both cases, it appears to be prudent to deploy genetic systems for intrinsic containment, confining the organisms’ survival to specific, defined environments. This can bridge the conflicting needs for risk reduction on the one hand and for convenience and cost-effectiveness of cultivation on the other. In contrast to restricting access to DNA of potential concern, genetic biocontainment systems largely require compliant actors, as the systems embedded in the host organism’s genome can be genetically inactivated. The notable exception is creating an orthogonal central dogma which would allow for semantically confining synthetic genes to synthetic host organisms with a genetic ‘firewall’ to natural organisms.

For research settings, current NIH guidelines list approved host-vector systems offering biocontainment with escape rates of less than 1 in 10^8^, lowering biosafety level (BL) requirements of work conducted with them. Synthetic genomics with increasingly versatile genome editing tools have brought about a shift from vector-based to genome-based work. To accommodate this, well-characterized genetic biocontainment systems controlling organism survival could similarly be approved for reduction of BL classification. Undoubtedly, widespread implementation of suitable genetic biocontainment systems can make work with biological agents much safer. As described above, there is already a variety of different systems available, many of which have orthogonal modes of action and can be combined to achieve containment much more stringent than NIH guidelines mandate. Achieving extremely stringent genetic containment will also play a pivotal enabling role for approval of field applications of GEMs, which will require control over cell counts that are several orders of magnitude higher than 10^8^. In addition to requirements regarding stringency and evolutionary stability, industrial-scale use cases will necessitate genetic biocontrol that is also cost-effective and environmentally unproblematic.

The rapidly changing landscape of synthetic genomics, enabling new capabilities and applications of engineering biological systems, requires advanced biosafety and biosecurity frameworks to ensure responsible research and commercial use. Thus, the continued development, review and adaptation of digital and biological safety mechanisms as major components in these frameworks is indispensable.
